# Supplementary material for: AgTx2-GFP, Fluorescent Blocker Targeting Pharmacologically Important Kv1.x (x = 1, 3, 6) Channels
Source: Toxins (Basel). 2023 Mar 18;15(3):229. doi: 10.3390/toxins15030229 (PMC10056440; doi:10.3390/toxins15030229)
Supplement: Supplementary file 1 [file toxins-15-00229-s001.zip › toxins-2251232-supplementary.pdf]

toxins

Article

AgTx2-GFP, Fluorescent Blocker Targeting Pharmacologically Important Kv1.x (x = 1, 3, 6) Channels

Alexandra L. Primak, Nikita A. Orlov, Steve Peigneur, Jan Tytgat, Anastasia A. Ignatova, Kristina R. Denisova, Sergey A. Yakimov, Mikhail P. Kirpichnikov, Oksana V. Nekrasova and Alexey V. Feofanov

Interaction of AgTx2-GFP with Kv1.3 channels on the membrane of HEK293 cells.

HEK293 cells (from the Russian collection of cell cultures, the Institute of Cytology RAS, Saint Petersburg, Russia) were grown in Dulbecco's modified Eagle's medium DMEM/F12 (Paneco, Russia) containing 5% fetal bovine serum (FBS, HyClone, Utah, USA) and 2 mM L-glutamine (complete medium). Transient transfection of cells with pmKate2-KCNA3-del was performed as described in [36] using GenJector-U reagent according to the manufacturer's protocol.

Confocal microscopy of HEK293 cells was performed as described in *Material and methods* secton.

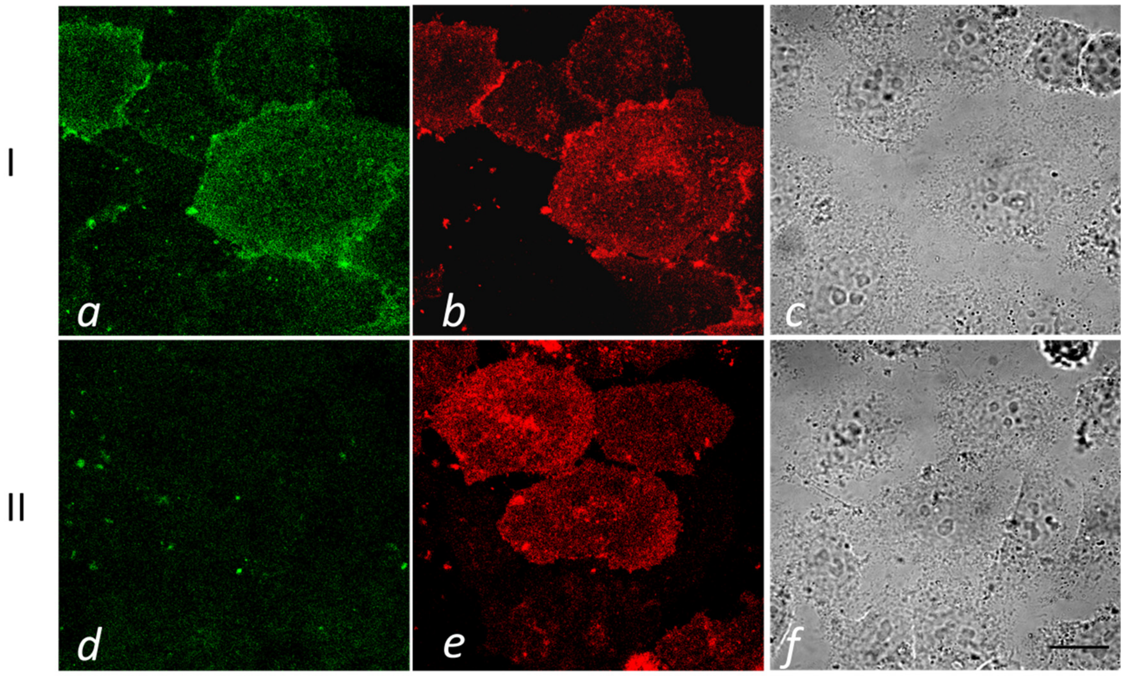

Figure S1. Confocal imaging of AgTx2-GFP interaction (green; *a*, *d*) with mKate2-Kv1.3 channels (red; *b*, *e*) transiently expressed in HEK293 cells. Confocal images were measured from the basal layer (plasma membrane) of living HEK293 cells. (rows I, II) Binding of AgTx2-GFP (40 nM, 20 min incubation) to mKate2-Kv1.3 (row I) and its displacement from the complexes (row II) by AgTx2 (40 nM AgTx2-GFP, 400 nM AgTx2, 20 min incubation). The scale bar is 20 µm. Panels (*c*,*f*) – transmitted light images of cells. .

AgTx2-GFP demonstrates no nonspecific binding on the membrane of cells in the absence of Kv1.3 channels.

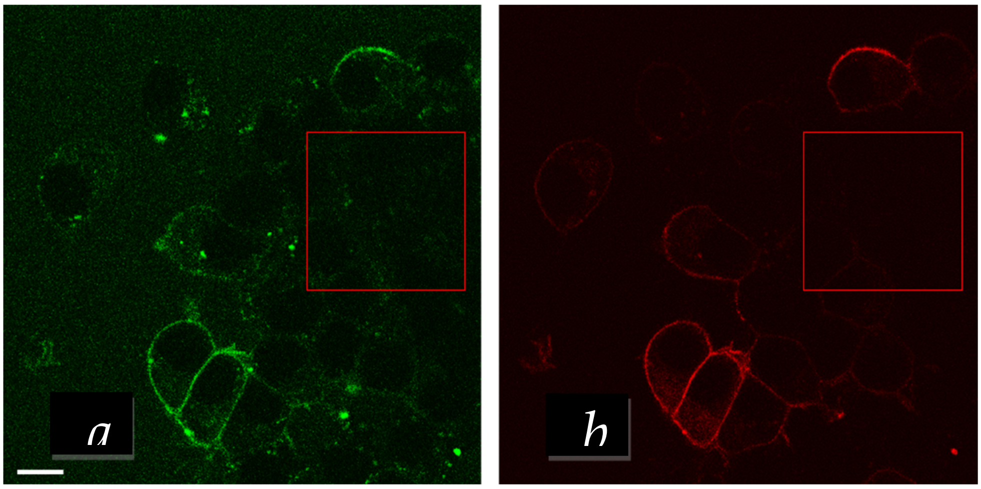

Figure S2. Confocal imaging of an interaction of AgTx2-GFP (8 nM, green) with Neuro 2A cells expressing mKate2-Kv1.3 (red). Red square marks a region with cells, which do not express mKate2-Kv1.3 (*b*) and demonstrate absence of nonspecific binding of AgTx2-GFP (*a*). Bar is 10 µm.

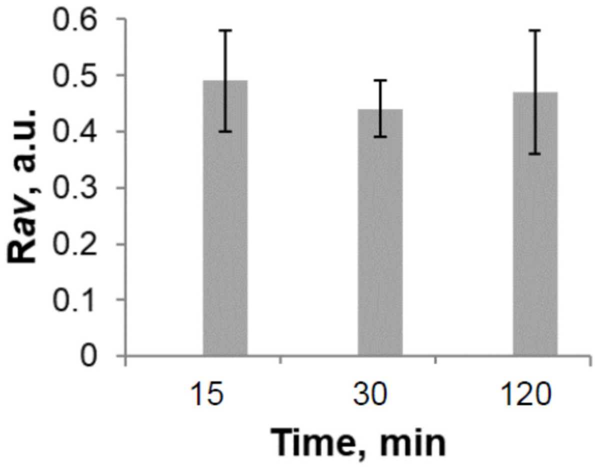

Figure S3. Time dependence of AgTx2-GFP binding to Kv1.3 channels on the membrane of Neuro 2A cells that was measured as the dependence of R_{av} (mean±SD) on the time of cells incubation with 8 nM of AgTx2-GFP.
